# The effect of forming implementation intentions on alcohol consumption: A systematic review and meta‐analysis

**DOI:** 10.1111/dar.13553

**Published:** 2022-09-29

**Authors:** Richard Cooke, Helen McEwan, Paul Norman

**Affiliations:** ^1^ School of Health, Wellbeing and Science Staffordshire University Stoke on Trent UK; ^2^ School of Psychology University of Worcester Worcester UK; ^3^ Department of Psychology University of Sheffield Sheffield UK

**Keywords:** alcohol, heavy episodic drinking, if‐then plans, implementation intentions, volitional help sheet

## Abstract

**Issues:**

Meta‐analysis was used to estimate the effect of forming implementation intentions (i.e., if‐then plans) on weekly alcohol consumption and heavy episodic drinking (HED). Sample type, mode of delivery, intervention format and timeframe were tested as moderator variables.

**Approach:**

Cochrane, EThOS, Google Scholar, PsychArticles, PubMed and Web of Science were searched for relevant publications to 31 March 2021. Random‐effects meta‐analysis was used to estimate the effect size difference (*d*) between individuals forming versus not forming implementation intentions on weekly consumption and HED.

**Key Findings:**

Sixteen studies were included in meta‐analyses. The effect size difference for forming implementation intentions on weekly alcohol consumption was *d*_+_ = −0.14 confidence interval (CI) [−0.24; −0.03]. Moderator analyses highlighted stronger effects for: (i) community (*d*_+_ = −0.38, CI [−0.58; −0.18]) versus university (*d*_+_ = −0.04, CI [−0.13; 0.05]) samples; (ii) paper (*d*_+_ = −0.26, CI [−0.43; −0.09]) versus online (*d*_+_ = −0.04, CI [−0.14; 0.06]) mode of delivery; and (iii) volitional help sheet (*d*_+_ = −0.34, CI [−0.60; −0.07]) versus implementation intention format (*d*_+_ = −0.07, CI [−0.16; 0.02]). In addition, effects diminished over time (*B* = 0.02, SE = 0.01, CI [0.03; 0.01]). Forming implementation intentions had a null effect on HED, *d*_+_ = −0.01 CI [−0.10; 0.08].

**Implications:**

Forming implementation intentions reduces weekly consumption but has no effect on HED.

**Conclusion:**

This review identifies boundary conditions on the effectiveness of implementation intentions to reduce alcohol consumption. Future research should focus on increasing the effectiveness of online‐delivered interventions and integrating implementation intention and motivational interventions.

## 1 INTRODUCTION

The World Health Organisation estimates that 5.3% of all deaths worldwide are attributable to alcohol consumption [1]. Consumption has been linked to increased likelihood of developing several cancers and liver disease [2, 3]. Heavy episodic drinking (HED), for example, men drinking more than five standard drinks or women drinking more than four standard drinks in a single session [4], has been linked to negative outcomes: blackouts, crime, injuries and sexually transmitted infections [5, 6]. Given the negative outcomes associated with alcohol consumption and HED there is an urgent need to identify effective interventions to reduce performance of these behaviours.

Asking people to form an implementation intention is an intervention that can be used to reduce alcohol consumption and HED. Implementation intentions identify a situational cue and link it to an appropriate behavioural response using an if‐then format; for example, *if* I am offered an alcoholic drink, *then* I will ask for a non‐alcoholic drink [7]. Forming an implementation intention facilitates identification of the critical cue specified in the *if* component and helps to automate the response specified in the *then* component of the plan [8]. Forming an implementation intention is associated with an average effect size difference of *d*_+_ = 0.59 for health‐related behaviours [9] and recent meta‐analyses show that forming implementation intentions increases physical activity [10], decreases dietary fat intake [11] and reduces smoking [12]. Malaguti et al. [12] report that forming implementation intentions reduces alcohol consumption, with an average effect size of *g* = 0.31.

However, there are four key limitations with Malaguit et al.'s meta‐analysis of the effect of implementation intentions on alcohol consumption, which justify the need for the current systematic review and meta‐analysis. First, their meta‐analysis was based on effect size differences reported across alcohol outcomes, that is, effect size differences for weekly drinking were pooled with effect size differences for HED. Such an approach lacks precision—it is unclear if implementation intentions reduce both weekly drinking and HED or if effects are limited to one outcome. Second, Malaguti et al.'s meta‐analysis did not include several recently published studies [13, 14, 15]. Third, effect size differences were calculated on follow‐up differences only, and did not account for group differences in baseline levels of consumption. Morris [16] notes several limitations with this approach. First, by only comparing consumption at follow‐up an apparent difference in consumption between the intervention and control groups may be illusory if the difference also existed at baseline. Second, if an intervention is effective, and those in the intervention group reduce their consumption, whereas those in the control group do not reduce their consumption, there will be greater variation in scores at follow‐up than baseline and, as a result, calculating the effect size difference based solely on follow‐up data is likely to underestimate the intervention effect due to a larger pooled standard deviation.

A fourth limitation with the Malaguti et al. [12] meta‐analysis is that they did not report any moderator analyses; it is unlikely that the effectiveness of implementation intentions will be constant across samples. Moderator analyses therefore help to identify the boundary conditions of any effects. The current paper considers the impact of four moderator variables. First, does *sample type* (community vs. university) affect the effect size difference? While university students represent a high‐risk group due to their harmful patterns of alcohol use [17], they may be more resistant to interventions to reduce their alcohol use [18] because alcohol is an integral part of their identity [19]. Second, does the *mode of delivery* (online, paper) impact the effect size difference? While online delivery can deliver interventions with greater reach, there is some evidence that this mode can result in low engagement with interventions [20, 21]. Third, does implementation intention *format* affect the effect size difference? Several formats have been tested in the alcohol domain: Implementation intentions (II) are if‐then plans that link a situational cue to a behavioural response, for example, *if* I am offered an alcoholic drink, *then* I will ask for a non‐alcoholic drink [14, 22, 23]; Mental contrasting implementation intentions (MCII) involve asking participants to link the most important inner obstacle to behaviour change to an action to overcome it [15]; Self‐affirming implementation intentions (SAII) identify a threat using an if‐then format (‘If I feel threatened or anxious, then I will …’) and present four options for addressing the threat (e.g., ‘I will think about things I value about myself’) [24, 25]; The volitional help sheet (VHS) involves linking situations that increase the urge to consume alcohol (e.g., ‘If I am tempted to binge drink when my friends push me to keep up with their drinking’.) with solutions to limit consumption (e.g., ‘Then I will seek out people who can increase my awareness about the problems of drinking’.) [26, 27, 28]. Finally, does the *time frame* between receipt of the intervention and follow‐up, impact the effect size difference? For example, there is some evidence that the effectiveness of online alcohol interventions decline over time [29].

### 1.1 Aims of the review

The primary aim of the present systematic review and meta‐analysis is to estimate the effect of forming implementation intentions on weekly alcohol consumption and HED by calculating the effect size difference in these outcomes separately between individuals asked to form versus not form implementation intentions. The secondary aim is to investigate the impact of sample type, mode of delivery, intervention format and time frame as moderators of effect size differences.

## 2 METHOD

### 2.1 Search strategy and inclusion criteria

The protocol for the systematic review was pre‐registered on the PROSPERO database, registration number CRD42017060628. Relevant studies were identified using the following methods: (i) electronic databases (Cochrane, EThOS, Google Scholar, PsychArticles, PubMed, Web of Science) were searched to 31 March 2021; (ii) reference lists of included articles were manually searched; and (iii) mailing lists of societies whose members have published research on the review topic (European Health Psychology Society, Kettil Bruun Society, UK Society for Behavioural Medicine) were used to request unpublished studies. The following keywords were used in the electronic searches: ‘implementation intentions’, and ‘alcohol’ or ‘binge‐drink*’. Searches generated 262 independent papers, after duplicates were removed. Papers were screened according to the following inclusion criteria:

1. Studies had to report results in English.
2. Studies had to report either weekly alcohol consumption and/or total number of HED episodes as an outcome(s).
3. Studies had to include at least one group of participants who were not asked to form an implementation intention (i.e., control) and at least one group of participants who were asked to form an implementation intention (i.e., intervention).
4. Studies had to report the sample size for both control and intervention groups and the mean and SD for the outcome variable(s), at both baseline and follow‐up, to allow for calculation of the effect size difference (*d*).

### 2.2 Selection of studies

**FIGURE 1:**
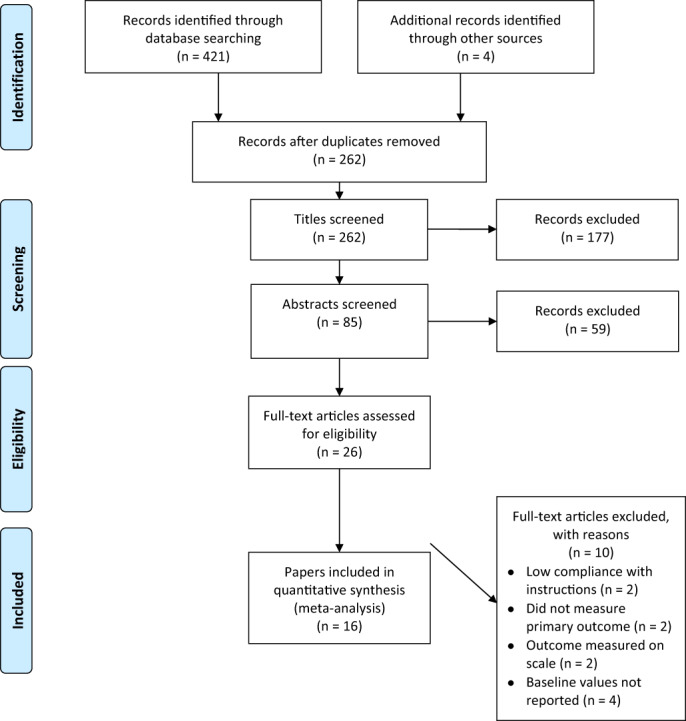
PRISMA study selection flowchart

Figure 1 provides a PRISMA flowchart outlining the eligibility and screening processes. The first two authors independently reviewed the titles and abstracts of the 262 papers for potential relevance to the research question. They excluded 177 papers at the title stage and a further 59 papers based on reviewing the abstracts against the inclusion criteria. Full text of potentially eligible papers (*n* = 26) was then assessed by both authors, according to the four inclusion criteria, with 10 papers excluded for one of four reasons: (i) two papers [20, 21] reported that few participants (<20%) formed implementation intentions to avoid binge drinking when asked to do so; (ii) two papers [30, 31] did not measure either outcome; (iii) in two papers [32, 33] participants were not asked to form implementation intentions; and (iv) in four papers [34, 35, 36, 37] mean and SDs were only reported at follow‐up, preventing calculation of effect sizes controlling for baseline values.

### 2.3 Assessment of methodological quality

The Cochrane Risk of Bias Tool [38] was used to assess the methodological quality of the included studies. This tool assesses bias in terms of random sequence generation, allocation concealment, blinding of participants and personnel, blinding of outcome assessors, completeness of outcome data reported, selective reporting and other bias. Papers were independently rated for methodological quality by the first and second authors. Overall quality for each study was determined through discussion between the first and second authors. Any disagreements were resolved through discussion. Risk of bias was classified in each study as low (all criteria graded low), moderate (one criterion graded high or two criteria graded unclear) or high (two or more criteria graded high or more than two graded unclear) [39].

### 2.4 Data extraction and coding

Data were independently extracted from included papers by the first two authors. Differences in data extraction were resolved following discussion. Where necessary the authors of included studies were contacted to obtain additional information. All authors who were contacted provided this information. Included studies were also coded for four moderator variables: (i) sample type (community, university); (ii) mode of delivery (online, paper); (iii) intervention format (II, MCII, SAII, VHS); and (iv) time frame (the number of weeks between intervention and follow‐up). Data files are publicly available (https://osf.io/jdz2x).

### 2.5 Data synthesis

This meta‐analysis is reported in accordance with the PRISMA statement [40]. Effect size differences for each study were calculated following Morris' [16] recommendation to control for baseline differences in outcome measures between intervention and control groups when calculating the effect size difference between outcomes at follow‐up. This is done by subtracting baseline mean values from follow‐up mean values for intervention and control groups, separately, and then using these new values to compute the effect size difference. Baseline SDs are used because they are expected to demonstrate less variance than follow‐up measures following the assumptions that: (i) the intervention and control groups are not expected to differ on the outcome at baseline; and (ii) *if* the intervention changes the outcome at follow‐up, variation in outcome scores is expected to be greater in the intervention compared to control group. An Excel spreadsheet was created to calculate effect size differences following Morris' formula. Effect sizes were imported into R and the metafor package [41] was used to calculate sample‐weighted average effect‐size differences (*d*_+_) based on a random‐effects model.

All meta‐analyses are reported in line with [42] where an effect‐size difference of *d* = 0.20 represents a small effect size, *d* = 0.50 represents a medium effect size and *d* = 0.80 represents a large effect size. Effect sizes were calculated such that negative values indicate greater reductions in alcohol consumption among intervention participants. Forest plots provide a graphical representation of the relative size of the effect size differences. Publication bias was assessed using funnel plots, Egger's Regression Test and Duval and Tweedie's Trim and Fill method. Homogeneity analyses were conducted using *Q* and *I* ^2^ statistics to determine whether variation in the effect size differences between samples was greater than chance; *I* ^2^ indicates the proportion of between‐study variance attributable to heterogeneity, where 25%, 50% and 75% are considered low, moderate and high values, respectively [43].

Categorical moderator variables were only tested when they were present and absent in at least four studies [44]. For categorical moderators, we calculated the *Q* homogeneity statistic separately for each category and then made comparisons to the overall *Q* statistic. Meta‐regression was used to estimate the effect of time frame as a continuous moderator. Mixed effects meta‐analysis was used to compare the effects of categorical moderator variables with time frame, the one continuous moderator variable.

### 2.6 Multiple outcomes, multiple samples and factorial designs

Where papers reported data on both outcomes data were extracted for both. Where papers recruited multiple samples [45], or split their sample [15], the effect size difference was calculated separately for each sample. Several studies [14, 23, 45, 46, 47, 48, 49] utilised fully factorial designs to test the effects of implementation intentions in combination with the effects of other types of intervention. For example, Hagger et al. [23] randomly allocated participants to one of four groups using a 2 (mental simulation; present vs. absent) × 2 (implementation intention; present vs. absent) factorial design. As a result, it was possible to extract two comparisons from this study that tested the effectiveness of implementation intentions: first, the comparison between the implementation intention only group and the control group; second, the comparison between the mental simulation plus implementation intention group and the mental simulation only group. In both cases, the only difference between the two conditions is the presence versus absence of implementation intentions. Using this approach allowed us to increase the number of comparisons included in the meta‐analysis, without compromising the independence of the data included. Table S1, Supporting Information, provides full details of these additional comparisons.

## 3 RESULTS

### 3.1 Study characteristics

Full details of the 16 included studies are provided in Table 1. Fourteen studies reported 27 comparisons of weekly consumption, while seven studies reported 20 comparisons of HED. Six studies reported using randomised controlled designs, four reported randomly allocating participants and one study mentioned that participants were randomised to condition. Two studies reported using factorial designs, one study reported using a cluster randomised controlled trial and one study employed a crossover design. Most studies were conducted in England (*k* = 12). Studies were also conducted in Australia (*k* = 1), Estonia (*k* = 1), Finland (*k* = 1), Switzerland (*k* = 1) and the United States (*k* = 2). Sample sizes for control and intervention groups ranged from 18 to 93. Percentage of female participants ranged from 40% to 100%. Mean age of samples ranged from 16.62 to 39.54 years. Regarding the representativeness of the samples, three studies [22, 24, 27] compared their samples to population level survey data to confirm that samples were similar in terms of demographic variables and consumption patterns. The remaining studies provided no information on the representativeness of their samples.

**TABLE 1:** Studies included in the review

Authors	Country	Sample	%F	Mean age	Mode of delivery	Follow‐up (weeks)	Intervention description	Control description	*d* (weekly)	*d* (HED)
Arden and Armitage [26]	England	University	66	20.57	P	2	*N* = 21; VHS	*N* = 18; VHS‐C	−0.34^a^	^c^
Armitage [22]	England	Community	53	38.40	P	4	*N* = 18; II	*N* = 21; AC	−0.14^a^	‐
Armitage [28]	England	Community^d^	83	33.78	P	4	*N* = 34; VHS	*N* = 31; VHS‐C	−0.24^a^	‐
Armitage and Arden [27]	England	Community	52	38.51	P	12	*N* = 18; VHS	*N* = 20; VHS‐C	−0.23^a^	‐
Armitage et al. [24]	England	Community	67	‐	P	4	*N* = 93; SAII	*N* = 93; HM	−0.77^a^	‐
Armitage et al. [25]	England	Community	55	17.09	P	8	*N* = 32; SAII	*N* = 35; HM	−0.19^a^	‐
Caudwell et al. [46]	Australia	University	74	20.86	O	4	*N* = 31; II *N* = 68; II	*N* = 30; HM *N* = 74; AS	‐ ‐	−0.19^a^ 0.20^b^
Ehret and Sherman [47]	USA	University	70	‐	O	2	*N* = 69; II *N* = 68; II	*N* = 69; HM *N* = 74; SA	−0.09^a^ −0.19^b^	‐ ‐
Hagger et al. [23]	England	University	58	20.32	O	4	*N* = 68; II *N* = 29; II	*N* = 81; MM *N* = 60; MS	−0.02^a^ −0.07^b^	0.24^a^ 0.05^b^
Hagger et al. [45]	England	University	88	19.72	P	4	*N* = 46; II *N* = 41; II	*N* = 39; MM *N* = 37; MS	−0.48^a^ −0.16^b^	−0.47^a^ −0.16^b^
	Estonia	University	70	20.83	P	4	*N* = 43; II *N* = 42; II	*N* = 47; MM *N* = 53; MS	−0.16^a^ −0.07^b^	0.03^a^ −0.06^b^
	Finland	University	64	23.66	P	4	*N* = 35; II *N* = 22; II	*N* = 30; MM *N* = 32; MS	0.19^a^ 0.09^b^	0.19^a^ 0.29^b^
Haug et al. [50]	Switzerland	Community	48	17.10	O	12	*N* = 66; II *N* = 70; II	*N* = 66; MM *N* = 70; MM	‐	0.12^a^ ^,^ ^e^ 0.00^a^ ^,^ ^f^
McGrath et al. [13]	England	Community	59	25.60	P	4	*N* = 42; VHS	*N* = 38; VHS‐C	−0.46^a^	
Norman and Wrona‐Clarke [48]	England	University	64	22.58	O	1	*N* = 58; II *N* = 53; II	*N* = 87; HM *N* = 85; SA	−0.26^a^ −0.25^b^	−0.17^a^ −0.34^b^
Norman et al. [49]	England	University	54	18.76	O	24	*N* = 74; II *N* = 82; II *N* = 80; II *N* = 81; II	*N* = 78; MM *N* = 84; SA *N* = 80; TPB *N* = 94; SA + TPB	0.15^a^ 0.16^b^ 0.13^b^ 0.20^b^	−0.02^a^ −0.01^b^ 0.12^b^ 0.17^b^
Norman et al. [14]	England	University	64	19.09	O	4	*N* = 44; II *N* = 30; II	*N* = 59; MM *N* = 63; TPB	−0.08^a^ 0.05^b^	−0.18^a^ −0.15^b^
Wittleder et al. [15] Low risk^g^	USA	Community	54	35.00	O	4	*N* = 48; MCII	*N* = 67; FT	−0.19^a^	–
Wittleder et al. [15] High risk^h^	USA	Community	54	35.00	O	4	*N* = 44; MCII	*N* = 41; FT	−0.43^a^	–

*Note*: *d* values are adjusted for baseline values for control and intervention groups.

Abbreviations: %F, percentage of sample that was female; AC, active control; AS, autonomy support; FT, filler task; HED, heavy episodic drinking; HM, health message; II, implementation intention; MCII, mental contrasting implementation intention; MM, mere measurement; MS, mental simulation; O, online delivery; P, paper delivery; SA, self‐affirmation manipulation; SAII, self‐affirmation implementation intention; TPB, theory of planned behaviour; VHS, volitional help sheet; VHS‐C, volitional help sheet control (see Table S2 for more details on control conditions).

^a^ Indicates that the effect size difference was calculated relative to a control group that received no intervention.

^b^ Indicates that the effect size difference was calculated relative to a control group who received a motivational intervention only. Further details are provided in Table S1.

^c^ Arden and Armitage (2012) measured HED on a seven‐point scale from 1 (never) to 7 (frequently).

^d^ All participants were smokers.

^e^ Participants received the intervention and then the control.

^f^ Participants received the control and then intervention.

^g^ Participants who scored <8 on the Alcohol Use Disorders Identification Test.

^h^ Participants who scored ≥8 on the Alcohol Use Disorders Identification Test.

### 3.2 Intervention characteristics

There were 19 university samples and 8 community samples. Online mode of delivery was used in 14 samples with paper delivery used in the other 13 samples. Nineteen samples asked participants to form an II, four samples completed a VHS, two samples completed a SAII and one sample completed a MCII. Studies used 1‐week (*k* = 2), 2‐week (*k* = 2), 4‐week (*k* = 12), 8‐week (*k* = 1), 12‐week (*k* = 2) and 24‐week (*k* = 1) follow‐up assessments. Variation in control conditions is summarised in Table S2.

### 3.3 Risk of bias

All included studies had a high risk of bias (see Figure S1). Ratings were driven by three domains: blinding of outcome assessor; incomplete outcome data; and allocation concealment. Blinding of outcome assessor was not reported in any of the studies. Incomplete outcome data, due to high rates of attrition, was rated as high or unclear risk in 10 studies. Allocation concealment was associated with unclear risk of bias in nine studies. Random sequence generation was reported in 12 studies and blinding of participants and personnel in 13 studies and was not possible in the study using a crossover design [50]. There was no evidence of selective reporting or other biases.

### 3.4 Meta‐analysis of forming implementation intentions on weekly alcohol consumption

**FIGURE 2:**
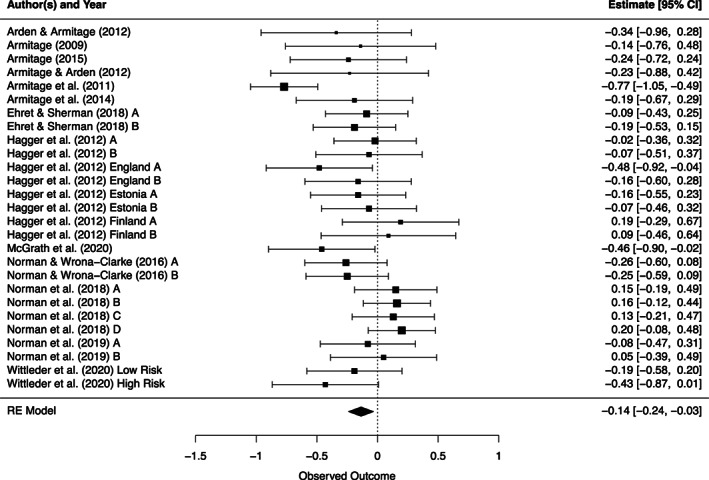
Forest plot of implementation intention intervention effectiveness in reducing the number of alcoholic drinks consumed per week. Samples denoted by the letter A represent a comparison between a control group and an implementation intention only group. Samples denoted by the letter B, C or D represent a comparison between a motivational intervention only group and a motivational intervention plus implementation intention group

Table 1 displays the effect size difference for the 27 comparisons of weekly alcohol consumption. The effect size difference for forming implementation intentions was *d*_+_ = −0.14 confidence interval (CI) [−0.24; −0.03] representing a significant effect. Figure 2 provides a forest plot of these results. A funnel plot was generated for this analysis but does not show evidence of publication bias (see Figure S3). Egger's regression test (*t*(25) = −0.44, *p* = 0.67), was non‐significant, and Duval and Tweedie's Trim and Fill method estimated that there are zero missing studies, providing additional evidence for a lack of publication bias. Results across studies were heterogeneous, *χ* ^2^(26) = 47.15, *p* = 0.01, *I* ^2^ = 45.09, so moderator analyses were conducted to try and account for this heterogeneity.

#### 3.4.1 Sample type

Results for community samples were significant, *d*_+_ = −0.38, CI [−0.58; −0.18], and homogenous, *Q*(7) = 10.00, *p* = 0.19, *I* ^2^ = 36.42, whereas results for university samples were non‐significant, *d*_+_ = −0.04, CI [−0.13; 0.05], and homogenous, *Q*(18) = 17.60, *p* = 0.48, *I* ^2^ = 8.57. A chi‐square test of these effect sizes indicated they were significantly different from one another (*Q*(1) = 12.66, *p* = 0.00).

#### 3.4.2 Mode of delivery

Paper delivery was associated with a significant effect size difference, *d*_+_ = −0.26, CI [−0.43; −0.09], whereas online delivery was not, *d*_+_ = −0.04, CI [−0.14; 0.06]. Both comparisons were homogenous (paper *Q*(12) = 20.89, *p* = 0.05, *I* ^2^ = 42.77; online *Q*(13) = 14.79, *p* = 0.32, *I* ^2^ = 17.42). A chi‐square test of these effect sizes indicated they were significantly different from one another (*Q*(1) = 5.07, *p* = 0.02).

#### 3.4.3 Intervention format

Due to a lack of studies testing MCII and SAII it was only possible to compare effect size differences for II and VHS. Completing a VHS had a significant effect on weekly consumption, *d*_+_ = −0.34, CI [−0.60; −0.07], and results were homogenous, *Q*(3) = 0.56, *p* = 0.90, *I* ^2^ = 0.00. In contrast, forming II had a non‐significant effect on consumption, *d*_+_ = −0.07, CI [−0.16; 0.02]. Results were homogenous. *Q*(18) = 16.91, *p* = 0.52, *I* ^2^ = 7.52. A chi‐square test of these effect sizes indicated they were significantly different from one another (*Q*(1) = 4.44, *p* = 0.04).

#### 3.4.4 Time frame

Meta‐regression was conducted to see if results were moderated by length of follow‐up. Time frame moderated results (*B* = 0.02, SE = 0.01, CI [0.03; 0.01]); as length of follow‐up increased, the effect of implementation intentions on consumption decreased.

#### 3.4.5 Mixed effects meta‐analysis

Mixed effects meta‐analysis was used to compare the effects of sample type and mode of delivery, with the effect of time frame; intervention format was not included because not all samples used II or VHS. The omnibus test for this analysis was significant (*Q*(3) = 31.20, *p* < 0.001), while the test of residual heterogeneity indicated a non‐significant amount of heterogeneity left to explain (*Q*(23) = 15.95, *p* = 0.86). There were significant effects for sample type, *B* = 0.29, SE = 0.10, CI [0.10; 0.48], *p* < 0.001 and timeframe, *B* = 0.01, SE = 0.00, CI [0.00; 0.02], *p* < 0.001; effects were greater among community samples and over shorter time frames.

### 3.5 Meta‐analysis of forming implementation intentions on HED

Forming implementation intentions had a null effect on HED, *d*_+_ = −0.01 CI [−0.10; 0.08]. Figure S2 provides a forest plot of these results, which were homogenous, *Q*(19) = 18.87, *p* = 0.47, *I* ^2^ = 5.84. There was no evidence of publication bias.

## 4 DISCUSSION

The present paper reports meta‐analyses of studies testing the effect of forming implementation intentions as an intervention to reduce weekly alcohol consumption and HED, after controlling for baseline differences in consumption. The sample‐weighted average effect‐size difference in alcohol consumption of forming implementation intentions was *d*_+_ = −0.14, representing a small, significant, effect size. By contrast, the effect size for forming implementation intentions on HED was null, *d*_+_ = −0.01. Overall, results provide modest support for the use of implementation intentions as an intervention to reduce weekly consumption, but no support for reducing HED.

Comparing results from the current meta‐analysis with those reported for physical activity, *d*_+_ = 0.31 [10], and eating a low‐fat diet, *d*_+_ = 0.49 [11], it is clear that the effect of forming implementation intentions on weekly alcohol consumption is considerably smaller, although, results for consumption are comparable to those reported by Black et al. [44] in their meta‐analysis of computer‐delivered alcohol interventions (*d* = 0.15). Forming implementation intentions may have had smaller effects on alcohol consumption compared to other health behaviours because consumption is often driven by contextual, cultural, environmental and social influences [51, 52, 53] and it is difficult for individuals to form implementation intentions that overcome these influences. Nonetheless, given their brevity, implementation intention interventions are likely to be low‐cost to deliver and, therefore, cost‐effective, despite their small effect on consumption [54].

Forming implementation intentions did not reduce HED. One explanation for the lack of effect of forming implementation intentions on HED may be the nature of the instructions used in studies. Fleig et al. [55] describe three key characteristics of plan enactment: specificity; instrumentality; viability. More specific plans are proposed to increase goal enactment because individuals who describe the anticipated behaviour and context precisely will be more likely to recognise the critical situation when it occurs. Plans that help achieve the desired outcome, such as those that focus on preparatory steps to action can be classified as instrumental. Finally, viability refers to successful goal enactment being more likely for individuals who have actual control over their behaviour, resources and opportunities. Fleig et al. tested the effects of these characteristics on plan enactment among a sample of patients seeking to increase their physical activity. Surprisingly, more specific behavioural responses resulted in lower plan enactment, suggesting that flexibility over behaviour is needed to bring about behaviour change. It is possible that the plans made by participants to avoid HED were too specific and inhibited flexibility to respond in potentially more effective ways that address cultural, contextual, environmental and social influences on consumption.

An alternative explanation for the lack of effect on HED is the current review only included tests of HED which recruited samples of university students or adolescents. Scott‐Sheldon et al. [56] reported that interventions targeting alcohol consumption in first year university students had only a trivial effect on HED (*d* = 0.07) so this could explain the lack of effect of forming implementation intentions on HED.

### 4.1 Moderator variables

Several variables were found to moderate the effect of forming implementation intentions on weekly consumption. The effect of forming implementation intentions was larger in community versus university samples. This suggests that implementation intentions are suitable for use in the general population, although further tests of the effectiveness of implementation intentions in community samples using online mode of delivery are needed as the current review was only able to identify one paper [15] that adopted this approach.

University students often resist attempts to reduce their alcohol consumption [18], given that excessive consumption is an integral part of many students' identities [19]. To overcome such issues, it may be necessary to combine implementation intention interventions with motivational interventions because planning interventions are less likely to produce behaviour change when motivation is lacking [57]. Support for interventions that target motivation and planning in combination has been found in the physical activity domain [58, 59, 60], however, studies that have tested interventions combining forming implementation intentions with motivational interventions have produced little evidence that this combination leads to greater reductions in consumption [61].

An area for future research would be to conduct studies to increase the synergistic effects of motivation and implementation intention interventions to reduce alcohol consumption would be to: (i) improve the integration of motivation and planning within a combined intervention; and/or (ii) split the delivery of the two elements. First, Ehret and Sherman [47] argue that effective integration of motivational and implementation intention interventions requires three factors to be present: (i) contextual flexibility; (ii) relative difficulty of the target behaviour(s); and (iii) personal relevance of the behaviour. Researchers can address the first two factors by asking participants to form more than one implementation intention. The third factor reflects the fact that participants must see alcohol reduction as personally relevant.

Second, all tests of the synergistic effects of motivation and planning, to date, have delivered both intervention components at the same time. An alternative approach would be to split the delivery of motivational and implementation intention intervention components. For example, The AlcoholEdu for College programme [62], used in many US universities, has a number of modules focusing on the risk of harmful drinking that are delivered before students start university and a planning task focusing on how to avoid harmful drinking that is completed when they are at university.

Forming an implementation intention on paper produced a significant effect size difference, whereas forming an implementation intention online did not. This comparison should be treated with caution, however, because there was a confound between mode of delivery and sample type; almost all community samples used paper as the mode of delivery whereas most studies that used online mode of delivery recruited university samples. Consequently, results for online delivery might underestimate the effect of forming implementation intentions because they were received by samples who are less motivated to reduce their consumption.

There was some evidence that completing a volitional help sheet led to greater reductions in weekly consumption compared to forming a traditional if‐then implementation intention. However, there are two caveats with this claim. First, only 4 volitional help sheet samples were included in this analysis, compared to 19 implementation intention samples. Second, 18 of the 19 implementation intention samples were recruited from university settings, while 3 of the 4 volitional help sheet studies recruited community samples.

Length of follow‐up moderated the effectiveness of implementation intentions for weekly consumption, although it should be noted that the longest follow‐up period was only 24 weeks. Chapman and Armitage [63] found that participants who completed booster implementation intentions—3 months after forming baseline implementation intentions—sustained increases in their fruit and vegetable consumption compared to those who formed implementation intentions without completing boosters. Booster implementation intentions might help to sustain the effects of forming implementation intentions on consumption. Research is needed to test the effectiveness of implementation intentions on consumption over extended periods of time (e.g., 12 and 24 months).

### 4.2 Gaps in the literature

Four important gaps in the literature on the effect of implementation intentions on alcohol consumption were identified. First, only five of the included studies recruited samples from outside of England. Studies testing the effectiveness of implementation intentions in a wider range of countries would help to confirm the generalisability of findings. Second, all but one study [50] recruited majority female samples. This means it is uncertain if current findings generalise to majority male samples; one study found that women reported consuming less alcohol on Friday night after completing a planning intervention, whereas men did not [31]. Moreover, Black et al. [44] note online interventions were more effective when the sample comprised more women. More research is needed to test interventions to reduce men's alcohol consumption especially as men consume more alcohol than women [64].

Third, few studies examined engagement with online implementation intention interventions. Although online delivery is viewed favourably by researchers, due to its greater reach, reduced costs and perceived preference among younger samples, if this mode of delivery compromises the effectiveness of the intervention through lack of engagement, then this is a serious concern. For instance, the current review excluded two studies [20, 21] as too few participants had formed an implementation intention. Given that engagement with online interventions is typically low [65], more research is needed on how to increase engagement as a means to increasing effectiveness. Finally, it is unclear if forming multiple plans is an effective approach to reduce alcohol consumption as only one study included in this review compared the effects of forming multiple plans to forming a single plan [27].

### 4.3 Strengths and weaknesses

The present systematic review and meta‐analysis has several strengths. First, it provides separate statistical estimates of the difference in weekly alcohol consumption and HED following formation of forming implementation intentions, showing that effect size differences are not the same across these outcomes. Second, it reports a meta‐analysis of the impact of implementation intention interventions to reduce alcohol consumption after controlling for baseline differences in consumption. Finally, it shows the effects of forming implementation intentions on consumption are moderated by intervention characteristics. Such findings identify the boundary conditions of implementation intention interventions and can inform future tests of implementation intentions to reduce consumption.

The current paper also has several weaknesses. First, consistent with concerns raised by other researchers [66], using the Cochrane Quality Appraisal tool to appraise experimental studies was challenging because this tool was developed to appraise randomised controlled trials (RCT). While RCT and experiments share properties, for example, randomising participants to condition, blinding participants and personnel to conditions, some aspects of quality routinely reported in papers using RCT designs (e.g., allocation concealment, blinding of outcome assessor) are not routinely reported in papers using experimental designs. As noted above, few of the included studies reported using randomised controlled designs and even those that did, were not conducted in the same way as RCTs. Second, one of the moderator analyses was based on a comparison group that only had four samples, a cut‐off based on a previous meta‐analysis of computer‐delivered alcohol interventions [44]. Even so, it was not possible to conduct some of the planned moderator analyses due to a lack of studies. Finally, alcohol consumption was assessed by self‐report in all studies. Tests of the effectiveness of implementation intention interventions using more objective measures of alcohol consumption, such as transdermal sensors worn round the ankle or wrist, that allow researchers to record consumption levels during a drinking event by measuring the presence of absence of biochemical markers of consumption, are needed [67].

## 5 CONCLUSIONS

This review identifies important boundary conditions on the effectiveness of implementation intention interventions to reduce alcohol consumption. Specifically, such interventions produce small but significant reductions in weekly consumption, but not HED, and are more effective when delivered to community samples and over shorter time frames. It should be noted that due to a lack of studies, and concerns about study quality, it is hard to draw firm conclusions about implementation intentions' effect on alcohol consumption. Future research should focus on how to increase the effectiveness of online implementation intention interventions, how to effectively combine implementation intention interventions with motivational interventions, and whether booster if‐then plans would help to sustain reductions in alcohol consumption.

## AUTHOR CONTRIBUTIONS

Richard Cooke conceived the review, wrote the review protocol, ran searches, completed data extraction and quality appraisal, ran the meta‐analyses and wrote the paper. Helen McEwan provided feedback on the review protocol, ran searches, completed data extraction and quality appraisal and provided feedback on the paper. Paul Norman provided feedback on the review protocol, provided guidance on the meta‐analytic approach and study coding and provided feedback on the paper.

## CONFLICT OF INTEREST

None to declare.

## Supporting information


**Table S1** Description of studies employing fully factorial designs
**Table S2**. Description of control conditions used in included studies
**Figure S1**. Risk of bias plots of ratings by domain and study
**Figure S2**. Funnel plot for weekly alcohol consumption
**Figure S3**. Forest plot for heavy drinking episodes. CI, confidence interval; RE, random effects
