# Supplementary material for: The effect of forming implementation intentions on alcohol consumption: A systematic review and meta‐analysis
Source: Drug Alcohol Rev. 2022 Sep 29;42(1):68–80. doi: 10.1111/dar.13553 (PMC10087331; doi:10.1111/dar.13553)
Supplement: Supplementary file 1 — Table S1 Description of studies employing fully factorial designs Table S2. Description of control conditions used in included studies Figure S1. Risk of bias plots of ratings by domain and study Figure S2. Funnel plot for weekly alcohol consumption Figure S3. Forest plot for heavy drinking episodes. CI, confidence interval; RE, random effects [file DAR-42-68-s001.docx]

Table S1. Description of studies employing fully factorial designs

|  | | | Comparisons | |
| --- | --- | --- | --- | --- |
| Authors | Study design |  | Intervention | Comparator |
| Caudwell et al. (2018) | 2 (implementation intentions: present vs. absent) ×2 (autonomy support: present vs. absent) | (A)  (B) | II  AS + II | Control  AS |
| Ehret & Sherman (2018) | 2 (implementation intentions: present vs. absent) × 2 (self-affirmation: present vs. absent) | (A)  (B) | II  SA + II | Control  SA |
| Hagger et al. (2012) | 2 (implementation intentions: present vs. absent) × 2 (mental simulation: present vs. absent) | (A)  (B) | II  MS + II | Control  MS |
| Hagger et al. (2012)^a^ | 2 (implementation intentions: present vs. absent) × 2 (mental simulation: present vs. absent) | (A)  (B) | II  MS + II | Control  MS |
| Norman & Wrona-Clarke (2016) | 2 (implementation intentions: present vs. absent) × 2 (self-affirmation: present versus absent) | (A)  (B) | II  SA + II | Control  SA |
| Norman et al. (2018) | 2 (implementation intentions: present vs. absent messages: present vs. absent) × 2 (self-affirmation: present vs. absent) × 2 (TPB messages: present vs. absent) | (A)  (B)  (C)  (D) | II  SA + II  TPB + II  SA + TPB + II | Control  SA  TPB  SA + TPB |
| Norman et al. (2019) | 2 (implementation intentions: present vs. absent) × 2 (TPB messages: present vs. absent) | (A)  (B) | TPB  TPB + II | Control  TPB |

*Note*.  ^a^ This comparison was used in separate samples recruited in England, Estonia and Finland. AS, autonomy support; II, implementation intentions; MS, mental simulation; SA, self-affirmation; TPB, theory of planned behaviour.

Table S2. Description of control conditions used in included studies

| Control condition | Description | Frequency | Reported by |
| --- | --- | --- | --- |
| Active control | Participants were asked to plan to reduce their consumption but not instructed how to do so. | 1 sample | Armitage (2009) |
| Filler task | Participants completed mental arithmetic tasks | 2 samples | Wittleder et al. (2020). |
| Health message | Participants were asked to read a message about the consequences of alcohol consumption | 5 samples | Armitage et al. (2011); Armitage et al. (2014); Caudwell et al. (2018); Ehret & Sherman (2018); Norman & Wrona-Clarke (2016) |
| Mere measurement | Participants only completed measures of alcohol consumption, demographic and psychological variables. | 9 samples | Hagger et al. (2012); Hagger, Lonsdale, Koka et al. (2012); Haug et al. (2020); Norman et al. (2018); Norman et al. (2019) |
| VHS control | Participants are given the VHS but not instructed on how to link actions to situations | 4 samples | Arden & Armitage (2012); Armitage (2015); Armitage & Arden (2012); McGrath et al. (2020) |

Figure S1. Risk of bias plots of ratings by domain and study.


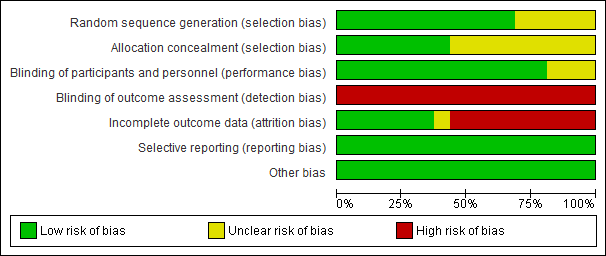


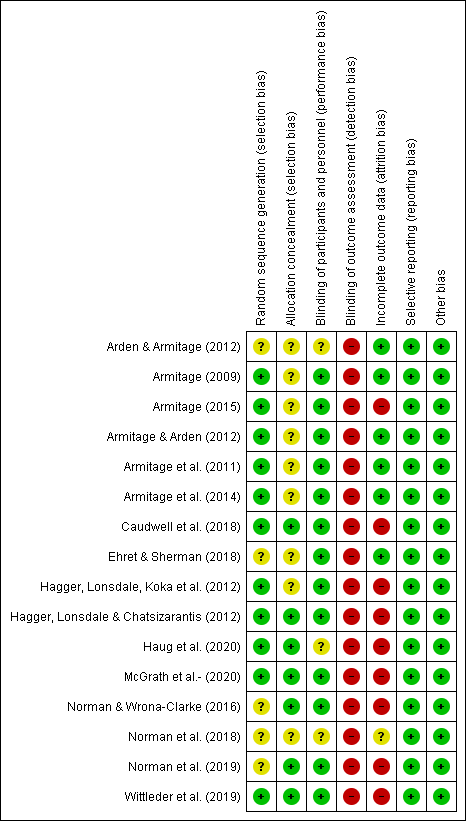


Figure S2. Funnel plot for weekly alcohol consumption.

Figure S3. Forest plot for heavy drinking episodes. CI, confidence interval; RE, random effects.
